# Eosinophil-mast cell pattern of intraepithelial infiltration as a marker of severity in CRSwNP

**DOI:** 10.1038/s41598-023-39149-8

**Published:** 2023-07-26

**Authors:** Matteo Gelardi, Rossana Giancaspro, Loren Duda, Vitaliano Nicola Quaranta, Cristina Pizzulli, Eugenio Maiorano, Filomena Milena Di Canio, Annamaria Ruzza, Lucia Iannuzzi, Nicola Antonio Adolfo Quaranta, Francesca Parisi, Michele Cassano, Andrea Marzullo

**Affiliations:** 1 Unit of Otolaryngology, Department of Clinical and Experimental Medicine, University of Foggia, Via Luigi Pinto 1, 71122 Foggia, Italy; 2 Unit of Pathology, Department of Clinical and Experimental Medicine, University of Foggia, Foggia, Italy; 3 Section of Respiratory Disease, Department of Basic Medical Sciences, Neurosciences and Sense Organs, University of Bari Aldo Moro, Bari, Italy; 4 Section of Pathology, Department of Emergency and Organ Transplantation (DETO), University of Bari “Aldo Moro”, 70124 Bari, Italy; 5 Otolaryngology Unit, Department of Basic Medical Science, Neuroscience and Sensory Organs, University of Bari Aldo Moro, Bari, Italy; 6 HNO Klinik, Kantonsspital Aarau, Aarau, Switzerland

**Keywords:** Biomarkers, Medical research, Pathogenesis, Risk factors

## Abstract

Chronic rhinosinusitis with nasal polyps (CRSwNP) is defined as a Type 2 eosinophilic disease, while CRSsNP is considered a Type 1 neutrophilic disease. Since neutrophils are also activated in eosinophilic CRSwNP, the eosinophil–neutrophil dualism has been revaluated. Among the inflammatory cells infiltrating sinus-nasal tissues, the role of mast cells (MCs) is not already recognized, although Clinical-Cytological Grading, which defines the severity of CRSwNP, attributes to mixed eosinophil-MC forms of CRSwNP a greater risk of recurrence. We aimed to examine nasal polyps from both a cytological and histopathological point of view, to evaluate the presence and localization of MCs. Cytological and histological examination of 39 samples of nasal polyps were performed. Immunohistochemistry was used to evaluate the presence of Tryptase + CD117 + MCs, which were counted both in the epithelial layer and in the lamina propria. A statistically significant correlation was found between intraepithelial MCs and CRSwNP severity (p < 0.001) and between the total eosinophil count and the total mast cell count (p < 0.001). Cytological examination and immunohistochemistry were comparable in detecting the presence of intraepithelial MCs (p = 0.002). The histological cut-off of 6 intraepithelial MCs was identified to detect severe CRSwNP (p < 0.001). MCs have been shown to be located in the *lamina propria* of almost all eosinophilic nasal polyps without significantly affecting their severity. Intraepithelial MCs are associated with greater severity of CRSwNP. Histopathological criteria of the eosinophil-MC form of CRSwNP in addition to the eosinophilic one, should be defined to guarantee patients effective and tailored treatments.

## Introduction

In the era of Precision Medicine (PM) research is focusing on the identification of the molecular pathways underlying Chronic rhinosinusitis (CRS), defined as a persistent sino-nasal inflammation associated with tissue remodelling, dysfunction of the sinuses' natural defence mechanisms, and exacerbation of different inflammatory patterns^1^.

EPOS 2020 Guidelines define three inflammatory patterns resulting from the epithelial barrier damage that underlies CRS: type 1 immune responses, targeting viruses; type 2 responses, targeting parasites; type 3 responses, targeting extracellular bacteria and fungi. Generally, these inflammatory patterns restore the barrier integrity and eliminate pathogens. However, in CRS, the epithelial barrier damage and the consequential penetration of exogenous agents results in a chronic inflammatory response that fails to resolve, but still typically utilizes the type 1, 2 or 3 pathways alone, or in combinations^2^.

In Western Countries, Type 2 inflammation is associated with 85% of Chronic rhinosinusitis with nasal polyps (CRSwNP), while Chronic rhinosinusitis without nasal polyps (CRSsNP) is mainly considered a Type 1 mediated disease^3,4^. Type 2 inflammation is characterized by eosinophilic activation and by increased levels of cytokines IL-4, IL-5 and IL-13. On the contrary, Type 1 and Type 3 responses are both characterized by neutrophilic activation, mediated by IFN-γ/IL-12 and IL-17/IL-22 respectively.

Recently, the eosinophil–neutrophil dualism has been revaluated, since neutrophils, as primary inflammatory cells, have been shown to be highly activated also in nasal polyps eosinophilic tissues^5^**.** Among the inflammatory cells that infiltrate the sinus-nasal tissue, the role of mast cells (MCs) is not already recognized, although a Clinical-Cytological Grading, already used to define the severity of CRSwNP as a function of comorbidities and cellular inflammatory infiltrate, attributes to mixed eosinophil-MC forms of CRSwNP a greater risk of recurrence^6^.

Based on this background, the study aimed to evaluate nasal polyps from a cytological and histopathological point of view, with the aid of immunohistochemistry, in order to evaluate the presence of mast cells, which can be assessed with nasal cytology but not with common stains used for histological examination.

## Materials and methods

Thirty-nine consecutive patients suffering from CRSwNP at the Department of Otolaryngology of the University Hospital of Foggia were recruited. Before undergoing endoscopic sinus surgery (EES), all patients underwent careful medical history evaluation, nasal cytology and nasal endoscopy. Then, during ESS, under general anesthesia, endoscopic-guided biopsy samples from nasal polyps were obtained Weil-Blakesley forceps.

Informed written consent was obtained from all participants. The study was approved by the Area 1 Ethics Committee (44/CE/2022 del 4.4.2022 e DCS n. 131 del 19.4.2022) of the University Hospital of Foggia. All experiments were performed in accordance with relevant named guidelines and regulations.

### Medical history

Before surgery, the complete medical history of each patient was collected, paying particular attention to atopy, asthma, aspirin and other nonsteroidal anti-inflammatory drugs (NSAIDs) sensitivity and previous polypectomy procedures. Moreover, each patient completed the Sino-Nasal Outcomes (SNOT)-22 survey to subjectively assess disease-related symptoms^7^.

### Nasal endoscopy

Preoperative nasal endoscopy was carried out by a 3.4 mm diameter flexible fiberscope (Vision-Sciences® ENT-2000) to assess the size of sinonasal polyps using Meltzer endoscopic polyp scores (Total Nasal Polyp Score, TNPS). According to this scoring system, each nasal cavity is scored as grade 0 (no polyps), grade 1 (polyp withinmiddle meatus), grade 2 (occupying the middle meatus), grade 3 (extending beyond the middle meatus, within the sphenoethmoid recess but not totally obstructing, or both) or grade 4 (completely obstructing the nasal cavity). Thus, the total score is the sum of left and right nostril scores, ranging from 0 to 8^8,9^.

### Nasal cytology and Clinical-Cytological Grading (CCG)

Cytological samples were collected before ESS by Nasal Scraping® (EP Medica, Italy), under anterior rhinoscopy, from the middle part of the inferior turbinate. Samples obtained were immediately smeared on a glass side, air-dried, stained with May-Grunwald-Giemsa (MGG) and then read at optical microscopy, with a 100× objective with oil immersion. Fifty fields were considered the minimum number to identify a sufficient number of cells. The predominant type of inflammatory cell was considered. Thus, four cytologic phenotypes were identified: neutrophilic, eosinophilic, MCs and mixed cellularity (eosinophil and MCs)^10^.

Thanks to nasal cytology findings and the medical history of each patient, the severity of CRSwNP can be defined. In particular, a Clinical-Cytological Grading (CCG) has been defined to assess the severity of CRSwNP and the related Prognostic Index of Relapse (PIR) according to both nasal inflammatory infiltrate and co-morbidities such as asthma, allergy and ASA sensitivity. The total CCG is given by the sum of the scores attributed to the phenotype, represented by each comorbidity, and to the endotype, represented by each inflammatory cell pattern. A CCG between 1 and 3 is considered low, 4–6 moderate and ≥ 7 high^11^.

### Histology and immunohistochemistry

The surgical specimens of nasal polyps were fixed in 10% buffered formalin and then subjected to usual processing by paraffin inclusion and hematoxylin–eosin (HE) staining.

Since MCs cannot be marked by HE, also immunohistochemical staining was performed. Indeed, as our intent was to investigate the occurrence and spatial distribution of mast cells in nasal mucosa, we excluded traditional histological stains, such as Toluidine blue and Giemsa, which allow to identify the intact MCs but not those in degranulation. Thus, we evaluated by immunohistochemistry the expression of two markers of MCs^12^: CD-117 (c-kit receptor), which is strongly expressed on the mast cell membrane and is not affected by the state of degranulation, to determine the presence of MCs and obtain the MC count^13^; Tryptase, which is considered as the marker of choice for the study of mast cell activation, to evaluate the degree of degranulation and, in a certain extent, mast cells activity ^14^.

Serial section 4 μm thick were cut from formalin-fixed paraffin-embedded tissue, deparaffinized in xylene, rehydratedin graded ethanol solutions, washed with distilled water and mounted on poly-l-lysine-coated glass slides.

Tryptase and CD117 expression was assessed by standard linked streptavidin–biotin horseradish peroxidase technique (LSAB-HRP) using specific monoclonal antibodies against Tryptase (mouse monoclonal primary antibody, clone G3) and anti-CD117 (rabbit monoclonal primary antibody, clone EP10) delivered by the BenchMark ULTRA (Ventana Medical Systems Inc, Roche Group).

Positive and negative controls were used. For each specimen, the whole section was examined under light microscopy (400× magnification), and the immunohistochemical expression was assessed as number of positive nuclei. In detail, ten high-power field (HPF) for each sample were recorded as mean value per HPF and both eosinophils and MCs were counted by two independent expert pathologists. Moreover, the presence of MCs both in the epithelial layer and in the lamina propria of the nasal mucosa was evaluated.

### Data analysis

All analyzes were conducted with SPSS-25 (SPSS, Chicago, USA). By means of the Kolmogorov–Smirnov test we verified that only age had a normal distribution while the other continuous variables had a non-parametric distribution. Continuous parametric variables were expressed as m (mean) ± SD (standard deviation). Continuous nonparametric variables were expressed as median (Interquartile Range (IQ) 25, 75). The dichotomous or non-continuous variables were expressed as%. Student's T test for independent samples was performed to compare the variables continue between subgroups.

For continuous variables with non-parametric distribution, the comparison for independent samples was performed by the Mann – Whitney U test. The Kruskal Wallis test was used for the non-parametric comparison of 3 groups and the ANOVA test for the comparison of continuous variables. The Chi-Square test was used for the comparison of the discontinuous variables.

A ROC curve was constructed to verify the accuracy of intraepithelial MCs in detecting the presence of high CCG. The cutoff was identified using the Youden index.

The Spearman correlation test was performed.

A significance value of p < 0.050 was assumed for all analyzes.

## Results

The study group was divided in three groups depending on CCG of CRSwNP (low, n = 10; medium, n = 13; high, n = 16). Patients’ demographic characteristics, comorbidities, CCG and SNOT-22 scores are summarized in Table 1. Table 2 shows nasal cytology and immunohistochemistry findings.

**Table 1:** Patients’ demographic features, comorbidities, CCG scores and SNOT-22 scores.

	Entire population	Low CCG, N = 10 (25.6%)	Moderate CCG, N = 13 (33.3%)	High CCG, N = 16 (41.0%)
Age, y m ± SD	53.51 ± 14.07	55.40 ± 15.15	54.92 ± 13.59	51.18 ± 14.35
Sex M, %	69.2	100	92.3	31.3
Allergy, %	56.4	0.0	46.2	100.0
Asthma, %	30.8	0.0	7.7	68.8
ASA sensitivity, %	33.3	0.0	15.4	68.8
CCG, count, median (IQ 25–75)	5 (4–8)			
SNOT 22, count, median (IQ 25–75)	13 (4–31)	14 (4–18)	11.5 (4.5–30.5)	16.5 (3–50)

**Table 2:** Nasal cytology and immunohistochemistry findings, subdivided by low, medium and high CCG.

	Entire population	Patients with low CCG, N = 10 (25.6%)	Patients with moderate CCG, N = 13 (33.3%)	Patients with high CCG, N = 16 (41.0%)	P value
Nasal cytology findings					
Patients with eosinophils, %	46.2	100.0	46.2	12.5	**< 0.0001**
Patients with mast cells, %	5.1	0.0	7.7	6.3	0.685
Patients with eosinophils + mast cells, %	48.7	0.0	46.2	81.3	**< 0.0001**
Immunohistochemistry findings					
Eosinophilis count, median (IQ 25–75)	22 (7–45)	15 (4–42)	17 (4.5–38.5)	30 (13.5–56.5)	0.234
Mast cells count, median (IQ 25–75)	11 (3–20)	9 (8–11)	6 (2.5–10.5)	17.5 (11–33)	**0.018**
Lamina propria mast cells count, median (IQ 25–75)	7 (3–16)	9 (3–11)	4.5 (2–8)	7 (4.5–18)	0.202
Intraepithelial mast cells count, median (IQ 25–75)	0 (4–9)	0 (0–5)	1 (0–4)	9 (3.5–22)	**< 0.0001**
Mast cells presence, %	97.4	90.0	100.0	100.0	0.226
Lamina propria mast cells presence, %	97.4	90.0	100.0	100.0	0.226
Intraepithelial mast cells presence, %	64.1	30.0	46.2	100.0	**< 0.0001**
Intraepithelial mast cells ≥ lamina propria mast cells, %	25.6	20.0	7.7	43.8	0.077

Significant are in value [bold].

No statistically significant correlations were found between MC distribution and laboratory data.

Charcot-Leyden Crystals (CLCs) were found in 10% of patients with low CCG, in 23% of patients with medium CCG and 31% of patients with high CCG.

No MCs were found in HE-stained samples.

**Figure 1:**
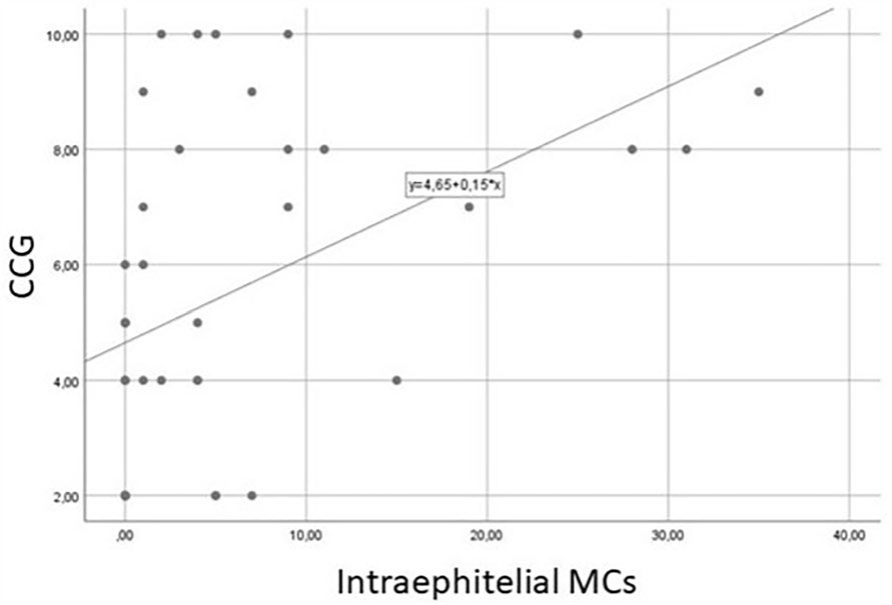
Spearman correlation between CCG and intraepithelial mast cells (R^2^:0.225; P < 0.001).

**Figure 2:**
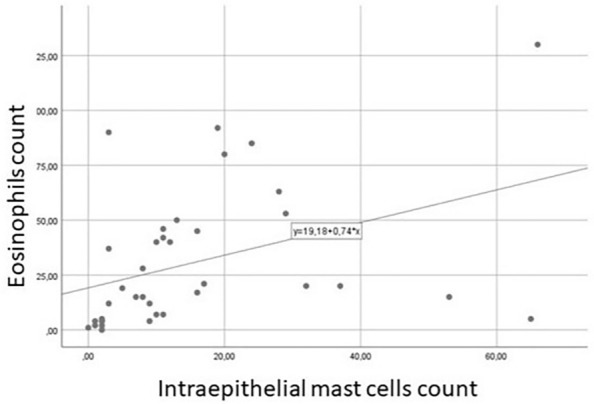
Spearman correlation between total MCs count and total eosinophils count (R^2^: 0.160; P < 0.001).

A statistically significant correlation was found between intraepithelial MCs and CCG (R^2^ = 0.225; p < 0.001) (Fig. 1), as intraepithelial MCs increased with increasing CCG, as well between Tryptase expression and CGG, as MC activity increased with CRSwNP severity. Similarly, a correlation was found between the total eosinophil count and the total mast cell count (R^2^ = 0.160; p < 0.001) (Fig. 2).

**Figure 3:**
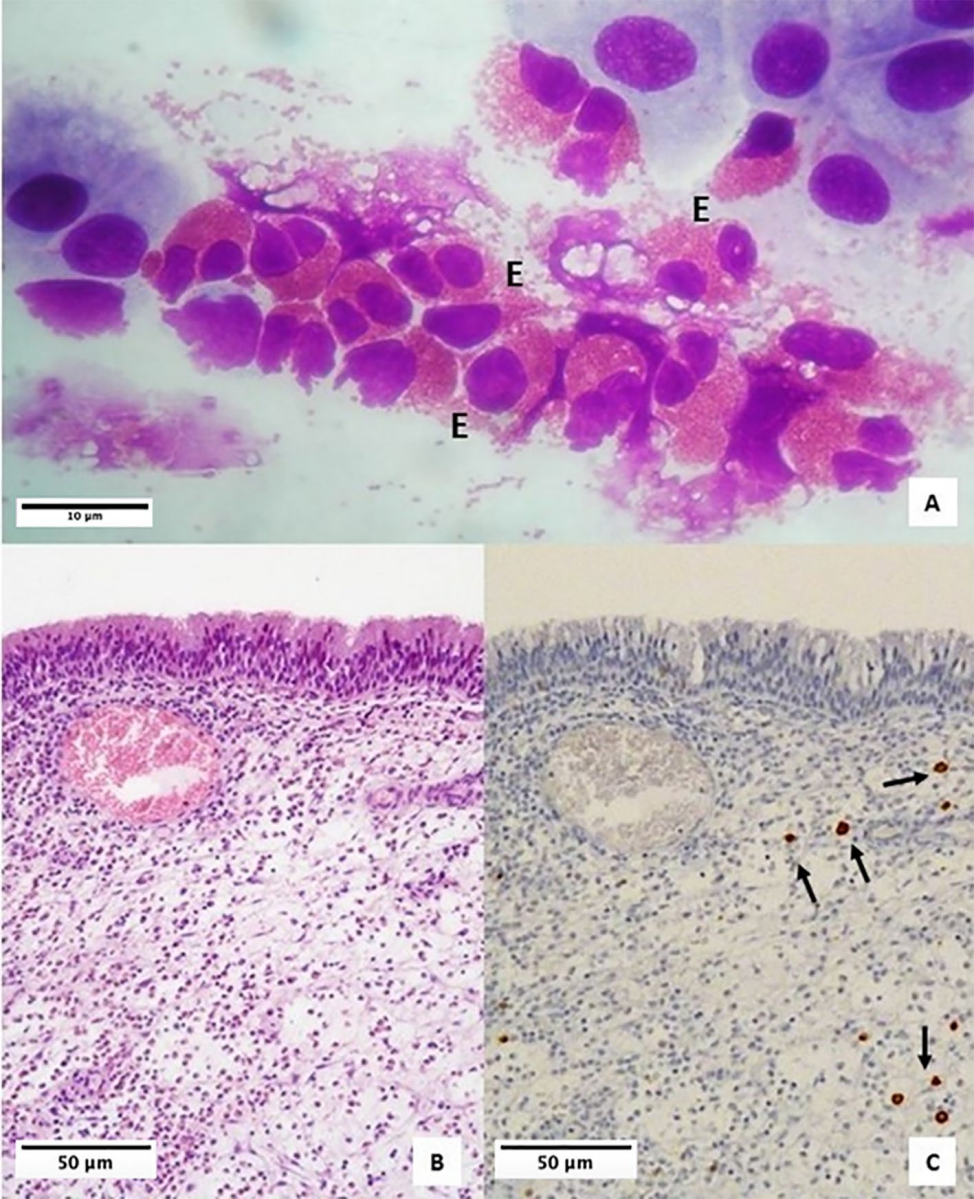
Samples from a patient with low CCG. (**A**) Nasal cytology. *E* eosinophil. MGG staining. Magnification ×1000. (**B**) Histology. HE staining. Magnification ×200. (**C**) Immunohistochemistry using specific monoclonal antibodies against anti-CD117. Magnification ×200.

**Figure 4:**
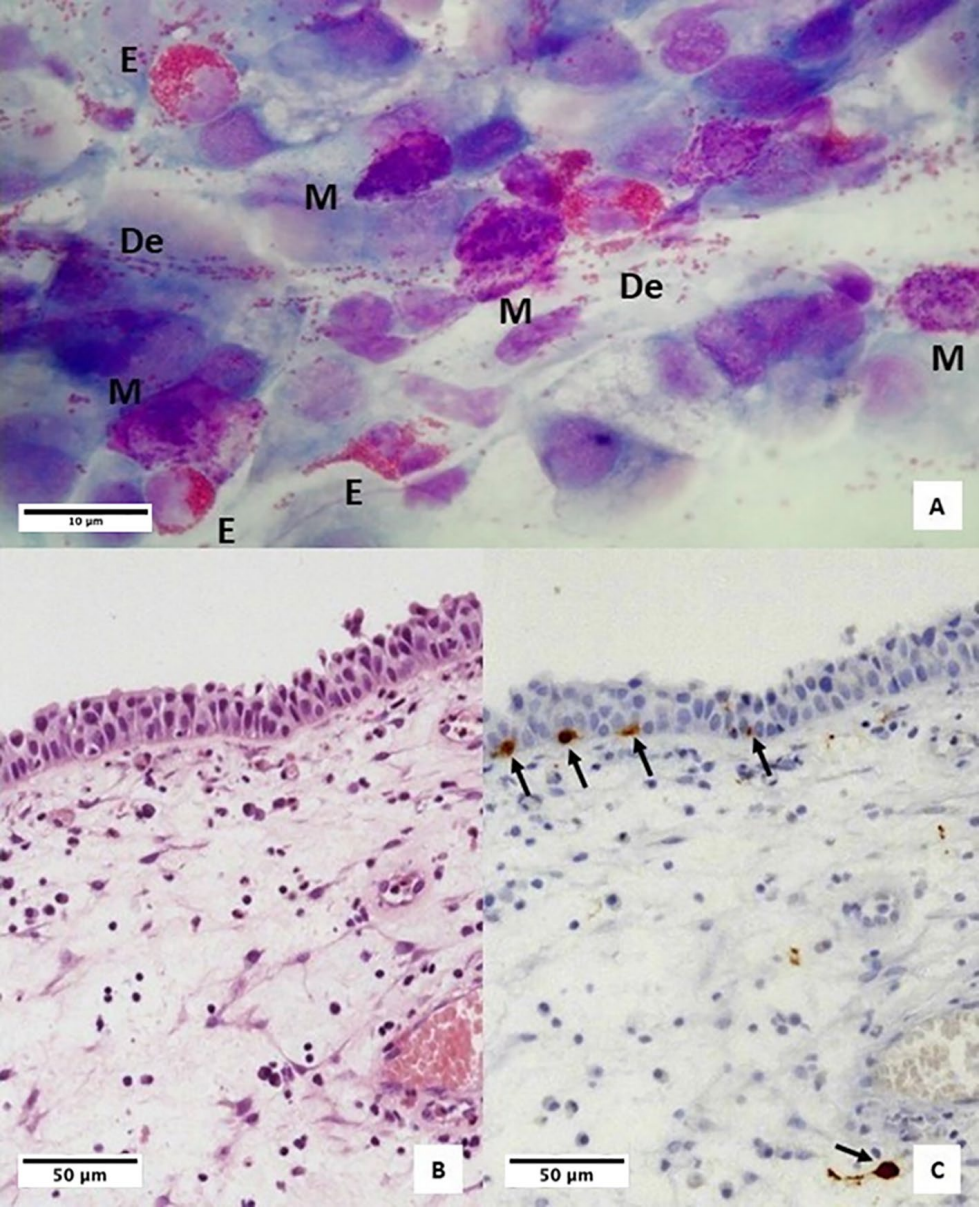
Samples from a patient with medium CCG. (**A**) Nasal cytology. *E* eosinophil, *M* mast cell, *De* degranulation. MGG staining. Magnification ×1000. (**B**) Histology. HE staining. Magnification ×200. (**C**) Immunohistochemistry, using specific monoclonal antibodies against anti-CD117, performed on the closest section to that used for histology. Magnification ×200. The arrows indicate CD117 + MCs.

**Figure 5:**
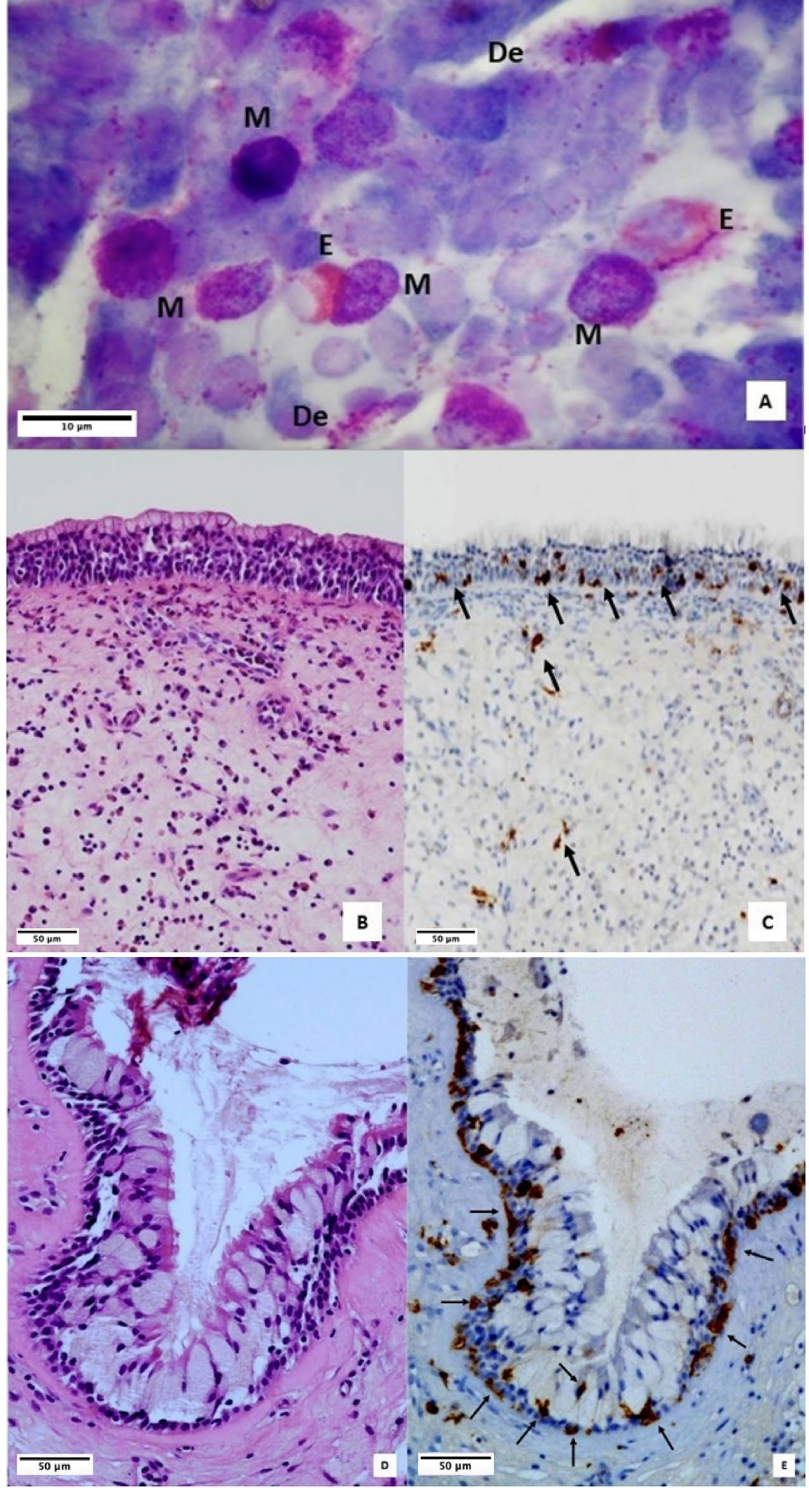
Samples from a patient with high CCG. (**A**) Nasal cytology. Magnification ×1000. (**B**) Histology. HE staining. Magnification ×200. (**C**) Immunohistochemistry using specific anti-CD117 monoclonal antibodies on the closest section to that used for histology. The arrows indicate CD117 + MCs. Magnification ×200. (**D**) Histology. HE staining. Magnification ×400 (**E**) Immunohistochemistry, using anti-CD117 monoclonal antibodies. Magnification ×400.

**Figure 6:**
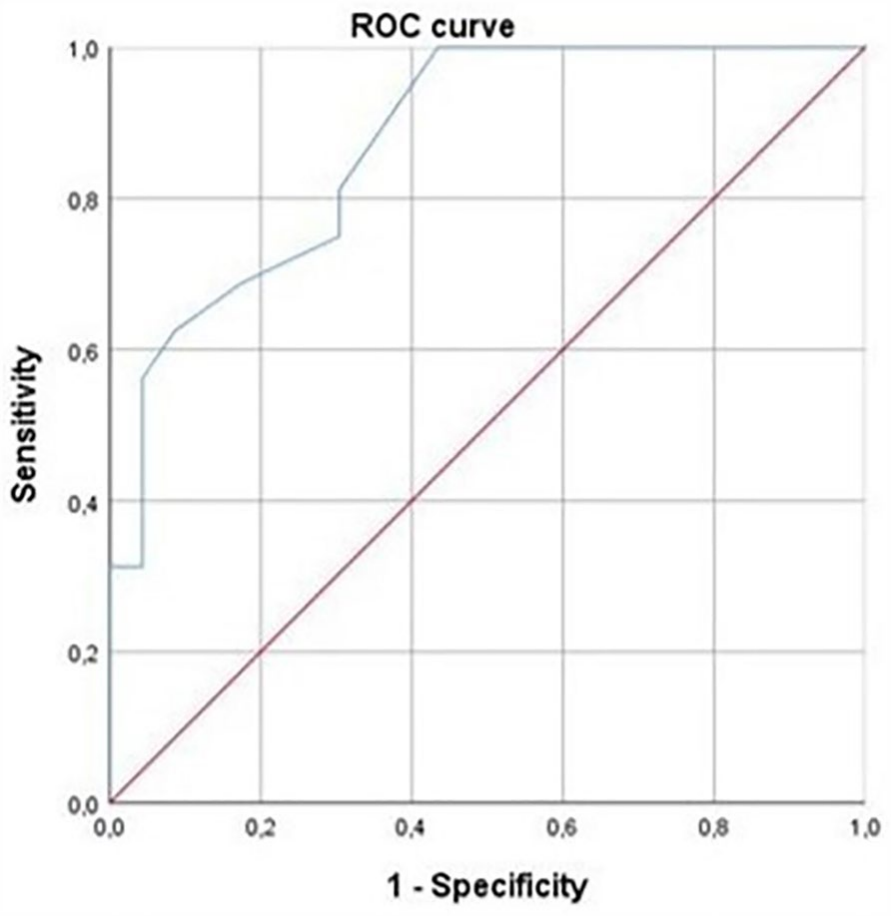
AUC: accuracy of intraepithelial mast cells in detecting the presence of high CCG. AUC: 0.874 (0.767–0.980), p < 0.001.

A statistically significant comparison was found between cytological examination and immunohistochemistry, as regards the histological presence of intraepithelial MCs and the finding of MCs at nasal cytology (p = 0.002) (Figs. 3A–C, 4A–C, 5A–E). Moreover, the histological cut-off of 6 intraepithelial MCs was identified to detect severe CCG. The cutoff of 6 had a sensitivity of 62.5%, a specificity of 91.3%, a positive predictive value of 83.3%, a negative predictive value of 77.7% (AUC = 0.874 (0.767–0.980), p < 0.001) (Fig. 6).

## Discussion

Type 2 CRS is the most extensively studied subset of CRS and, thus, many of the molecular details have been confirmed at the protein level. As a matter of fact, the first data date back to almost 20 years ago, when Van Zele and colleagues described a Th2 polarization in CRSwNP, once defined as nasal polyposis (NP), characterized by abundant eosinophils, high IL-5 levels, and IgE formation. CRSsNP instead was described as Th1 polarized disease, with high levels of IFN-c and TGF-b^15^.

Since then, several studies have focused on the dualism between Type 1 and Type 2 inflammation, not considering Type 3 inflammation, which has not yet been extensively studied^16^. Up to now eosinophilic-neutrophilic inflammation has been considered as black and white, almost implying mutual exclusion in CRS. Recently, this dualism has been revaluated, as it trivializes much more complex cellular and cytokines interactions in Type 2 inflammation, which displays also a severe neutrophilic inflammation^17^. As a matter of fact, neutrophils are traditionally considered primary inflammatory cells, as they are constant defenders and first cellular responders to tissue injury and infection, responsible for maintaining tissue homeostasis^18^. Since the occurrence of polyps depends on an exaggerated inflammatory reaction, it is not surprising that the inflammatory infiltrate of patients with severe and relapsing forms of CRSwNP, which is considered the inflammatory disease par excellence, may be characterized by abundant neutrophils. In addition, eosinophilic activation is traditionally associated with the presence of Charcot-Leyden crystals (CLCs), the crystallized form of Galectin-10, which is a major constituent of the cytoplasm of eosinophils that characteristically forms bi-piramidal hexagonal crystals when eosinophils are intensely activated and undergo ETosis, causing a crystallopathy^19^. CLCs have been shown to trigger epithelial cells to recruit neutrophils and produce neutrophil-activating cytokines, such as GM-CSF, TNF-a, and IL-1, that could potentially prime neutrophil effector function^20^. Actually, a recent study has not only shown that CLCs colocalize strongly with Gal-10 + CD15 + eosinophils and Gal-10 + Tryptase + mast-cells within the lamina propria, but also the strong correlation between the expression of Gal-10, the eosinophilic-mast cell infiltrate and severity of CRSwNP, according to the Clinical-Cytological Grading (CCG)^21^. Indeed, already in 2009 a CCG based on endotype (inflammatory infiltrate to nasal cytology) and phenotype (comorbidities such as asthma, ASA-sensitivity and allergies) was proposed to establish the severity of CRSwNP and the related Prognostic Index of Relapse (PIR)^22^. According to the CCG, the eosinophil-mast cell endotype had already been proposed as the most difficult to treat, with the greatest risk of recurrence. The aforementioned study, conducted with immunofluorescence and confocal laser microscopy has confirmed the involvement of MCs in the pathogenesis of the spectrum of Type 2 diseases, such as CRSwNP. In particular, MCs could secrete type 2 cytokines (IL-4, IL-5, IL-13), facilitating eosinophilic inflammation and causing tissue remodeling through the degradation of the extracellular matrix. Therefore, greater pathophysiological dignity should be conferred on MCs and even eosinophilic inflammation should be better defined as mixed eosinophilic-MC inflammation.

As proof of this, as shown in the results, the histological examination of HE-stained specimens did not reveal the presence of MCs in any sample. However, immunohistochemical evaluation revealed the presence of MCs within the *lamina propria* in 97.4% of cases and within the epithelial layer in 61.4% of cases. Moreover, intraepithelial MC count increased statistically significantly with increasing severity of CRSwNP, according to the CCG and, more in detail, the cut-off of 6 intraepithelial MCs was identified to detect high CCG.

Since eosinophils were found in all analyzed samples, almost all eosinophilic forms of CRSwNP, traditionally considered as Type 2-eosinophilic forms, showed MCs. However, in most cases, the MCs were located at the *tonaca propria* layer. As the HE staining does not allow to highlight MCs, the latter cytotypes are not described in common histological examinations.

Moreover, this subepithelial localization explains why the nasal cytology also described several eosinophilic forms, without mentioning MCs. In fact, nasal cytology, contrary to what is typically believed, is a non-invasive diagnostic tool that involves sampling by scraping, which is a bloodless procedure, therefore limited to the epithelial level. Indeed, by definition, nasal cytology does not allow to evaluate subepithelial cells but only intraepithelial and infiltrating cells. On the contrary, nasal cytology proved to be a valid tool for identifying cases with intraepithelial MCs, as a statistically significant correlation was found between cytological examination and histological immunohistochemical examination, in detecting intraepithelial MCs.

Interestingly, intraepithelial MCs were directly proportional to CCG and, thus, to the severity of CRSwNP. Indeed, although MCs have always been considered as “children of a lesser god”, these cells MCs likely play a central role in orchestrating immune responses at barrier tissues. As a matter of fact, the characterization of MCs phenotypes in terms of functional heterogeneity has been traditionally underestimated, especially in a setting of mucosal inflammation^23^. Only recently, several studies have shown that two major human MCs subtypes exist: sub epithelial MCs (MC_TC_), expressing both tryptase and chymase in conjunction with cathepsin G and carboxypeptidase A3 (CPA3), and mucosal epithelial MCs (MC_T_), expressing only tryptase^24^. In CRSwNP and asthma, there is a prevalent expansion of intraepithelial MC_T_ expressing CPA3 and of MC_TC_ infiltrating airway smooth muscle^25^. These subtypes are responsible for the production of cytokines that not only activate eosinophils but also directly promote the degradation of the extracellular matrix, generating a vicious circle thanks to which eosinophils and MCs attract and activate each other causing exacerbation of inflammation and epithelial damage^26^. This explains the correlation between total MC and eosinophil count shown in the results, as the two cytotypes influence each other and increase as the severity of CRSwNP increases^27^. Increased levels of these two cytotypes are also responsible for the increased production of CLCs, which has a correlation with CRSwNP severity. The formation of CLCs is strongly associated with disease severity, since it can induce a crystallopathy by the activation of NLRP 3 inflammasome and the stimulation of innate and adaptative immunity^28^. This crystallopathy, together with the release of lytic enzymes such as major basic protein (MBP), tryptase and collagenase by both eosinophils and MC induces an exfoliation of epithelial cells and the rupture of desmosomes, allowing pathogens and other exogenous substances to penetrate the mucosal barrier^29,30^. Among pathogens, *Staphylococcus aureus*, traditionally associated with the pathogenesis of CRSwNP, has been shown to replicate within the MCs of the nasal mucosa and ultimately induces lysis of MCs and release of intracellular content^31,32^. As evidence of this, among the histological samples analyzed, those of patients with high intraepithelial MC infiltrate showed a variable de-epithelialization severity (Fig. 6).

The activation of MCs has also been proven by the statically significant correlation between Tryptase and CCG: the expression Tryptase, which is considered a marker of mast cell degranulation, increased with increasing CCG. Indeed, the degranulation of mast cells leads to the release of preformed mediators, such as tryptase, chymase, carboxypeptidase, histamine, and heparin, that in turn cause the release of de novo synthesized mediators such as lipid mediators, chemokines, growth factors and interferons, which exacerbate the disease. Tryptase, in particular, is a neural serine protease that is stored in secretory granules and released following mast cell activation by IgE-dependent and independent processes^33^. This protease is associated with the development of allergic and inflammatory responses, that are directly associated with tissue remodeling, due to the many biological effects of Tryptase, including the inactivation of fibrinogen, fibronectin, collagens, and lipoproteins, regulation of mesenchymal cell proliferation and survival, upregulation of adhesion molecules, and induction of growth factors and cytokine^34^. Moreover, it is noteworthy that Tryptases activates circulating eosinophils, inducing the release of granule-associated enzymes and, consequently, generating a vicious circle^35^.

**Figure 7:**
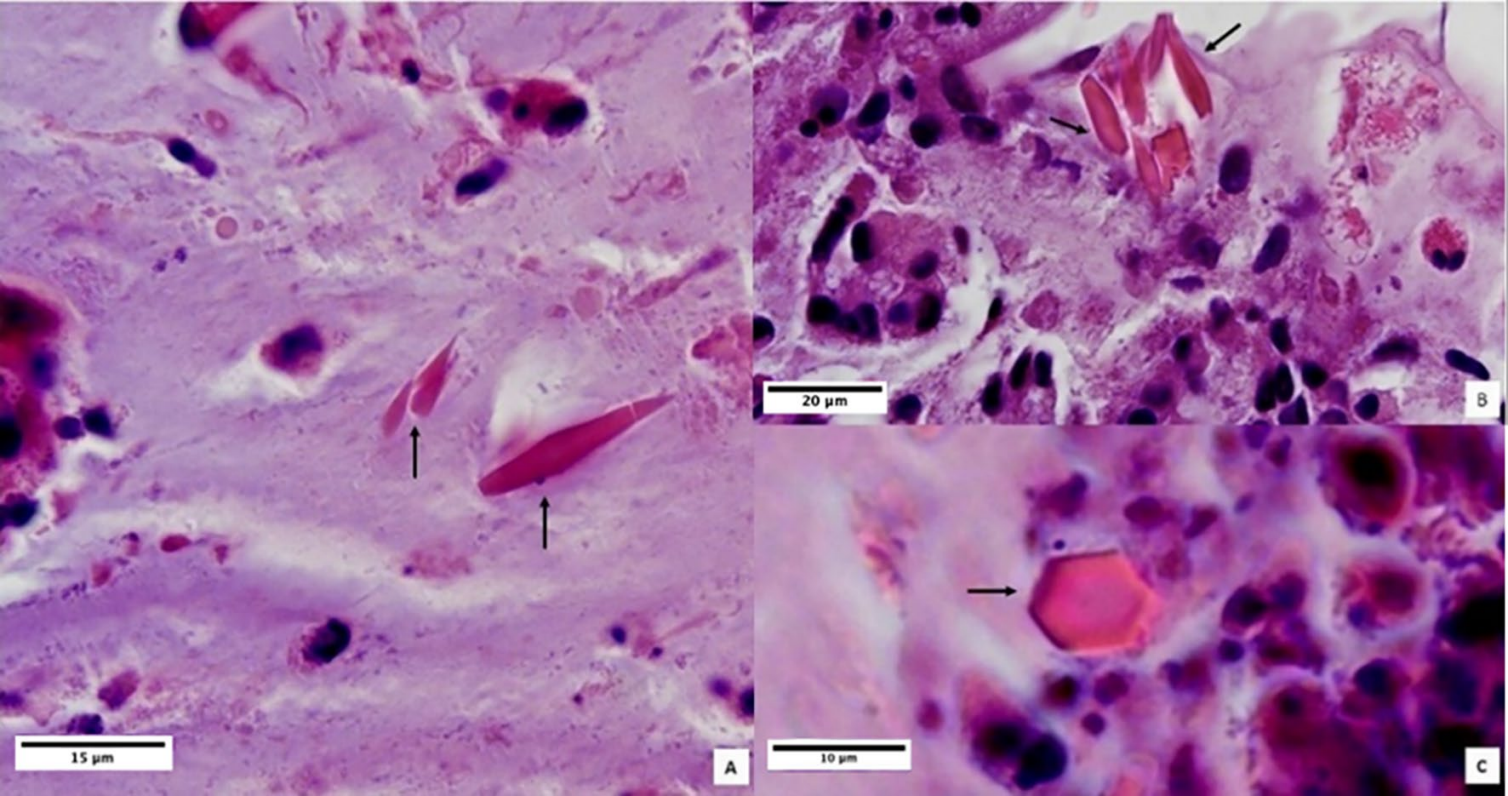
Eosinophilic mucin with Charcot-Leyden crystals (CLC) inside, single (**A**) or associated with each other (**B**), indicated by the arrows. (**C**) Cross-section of a single CLC showing the hexagonal shape. HE staining. Magnification ×1000.

Since MCs and eosinophils appear to influence each other, it is unclear whether CRSwNP with MC epithelial infiltration represent an evolution of the most severe eosinophilic forms of CRSwNP or it could be considered a stand-alone form of CRSwNP, characterized by abundant CLCs, being therefore more difficult to treat and more prone to relapse^36^ (Fig. 7). Nevertheless, in light of these evidences, the role of MCs in CRSwNP pathophysiology should be re-evaluated and emphasized^37,38^.

In this context, we believe that the current classification of CRS inflammatory patterns into Type 1, Type 2 or Type 3 should be revised. In particular, Type 1 and Type 3 endotypes, both characterized by predominant neutrophilic infiltrate, could be considered as subgroups of a single type, Type 1 precisely, which is characterized by neutrophils. These neutrophilic forms of CRSwNP, mainly associated with CRSsNP or CRSwNP with low PIR, could be subclassified into Type 1A and Type 1B depending on the absence (previously defined as Type 1) or presence of IL-17 (previously defined as Type 3), respectively (Table 3).

**Table 3:** Cytokines and enzymes of each type of inflammatory pathway.

Type 1		Type 2	Type 3
1A	1B		
IFN-γ	IL-17A	IL-4	IL-4
TNF-α	IL-22	IL-5	IL-5
IL-1β	IL-23	IL-9	IL-9
IL-6	IFN-γ	IL-13	IL-13
IL-8	TNF-α	ECP	CD117
	IL-1β		Tryptase
	IL-6		Chymase
	IL-8		Carboxypeptidase
			ECP

On the other hand, Type 3 should refer to the eosinophilic-MC forms of CRS, characterized by both abundant eosinophils and intraepithelial MCs, which are typically associated with nasal polyp formation and a high relapse rate. Indeed, while mast cells have been shown to be located in the *lamina propria* of almost all types of nasal polyps without significantly affecting their severity, their intraepithelial localization determines a greater severity of CRSwNP, according to the CCG.

The concept of intraepithelial infiltration could be used precisely to reformulate the diagnostic criteria of CRSwNP and of its several subtypes, as is already the case for celiac disease or eosinophilic esophagitis (EoE). As a matter of fact, EoE is defined as esophageal dysfunction in the presence of > 15 intraepithelial eosinophils per high-power field (eos/HPF) in either the mid or distal esophagus^39^. Indeed, the current focus of EoE pathologic evaluation is the peak eosinophil count (PEC), although histologic features other than eosinophilic inflammation are also commonly observed. Moreover, reduced PEC is considered a universal goal in clinical management and constitutes an endpoint in clinical trials of therapies for EoE^40^. Similarly to EoE, the diagnosis of coeliac disease (CD) is based on several criteria including positive serology, a spectrum of duodenal damage and clinical symptoms and/or risk conditions, and response to a gluten-free diet (GFD) in patients bearing the HLA-DQ2 or DQ8 genotypes. Among the histopathological changes, duodenal intraepithelial lymphocytes (IEL) count is considered a useful tool for CD diagnosis^41^. In particular, 25–29 IEL/100 enterocytes are considered borderline and > 30 IEL/100 enterocytes are considered diagnostic for CD^42,43^.

Since CRSwNP has been shown to be characterized by intraepithelial infiltration of both eosinophils and MC, the introduction of their count in the pathology report could be useful and cheap diagnostic tool to characterize nasal polyps and possibly define their severity. Furthermore, since intraepithelial MC are associated with de-epithelialization, the reduction of their count could represent a goal for treatment management.

## Conclusion

Further studies are needed to understand whether massive eosinophilic inflammation is responsible for the recall of mast cells at the intraepithelial level or if other factors contribute to the recruitment of MCs at the intraepithelial level. A correct definition of the mixed eosinophil-MC CRSwNP form could have important therapeutic implications, especially after the introduction of biological treatments alongside existing treatments in the management of chronic rhinosinusitis with nasal polyps^44^. In fact, although biological treatments are now considered valuable therapeutic tools for the management of CRSwNP, only a careful choice of patients suitable for treatment allows to obtain the maximum therapeutic benefit and to reduce the economic burden. In this sense, a close alliance with pathologists is also fundamental, to establish the histopathological criteria that define the eosinophil-MC form of CRSwNP, as well as to possibly confirm the cut-off of intraepithelial MCs associated with greater severity of CRSwNP.

## Data Availability

Data available on request from the corresponding author.
